# Anti-Angiogenic Effect of Metformin in Mouse Oxygen-Induced Retinopathy Is Mediated by Reducing Levels of the Vascular Endothelial Growth Factor Receptor Flk-1

**DOI:** 10.1371/journal.pone.0119708

**Published:** 2015-03-18

**Authors:** Soo Geun Joe, Young Hee Yoon, Jeong A Choi, Jae-Young Koh

**Affiliations:** 1 Department of Ophthalmology, Asan Medical Center, University of Ulsan College of Medicine, Seoul, Korea; 2 Department of Neurology, Asan Medical Center, University of Ulsan College of Medicine, Seoul, Korea; 3 Neural Injury Research Center, Asan Institute for Life Sciences, University of Ulsan College of Medicine, Seoul, Korea; University G. D'Annunzio, ITALY

## Abstract

**Purpose:**

To evaluate the effect of metformin on vascular changes in oxygen-induced retinopathy (OIR) in mouse, and to elucidate the possible underlying mechanism.

**Methods:**

OIR mice were treated with metformin by intraperitoneal injection from postnatal day 12 (P12) to P17 or P21. At P17 and P21, vessel formation and avascular areas were assessed using retinal flat mounts. Levels of vascular endothelial growth factor (VEGF) were measured by enzyme-linked immunosorbent assays, and the effects of metformin on VEGF-induced proliferation of human umbilical vein endothelial cells (HUVECs) were assessed. The effects of metformin on the levels of Flk1 (VEGF receptor-2) and phosphorylated Flk1 (pFlk1) were measured by Western blotting (HUVECs) and immunohistochemistry (retinal tissue).

**Results:**

Retinal morphologic changes were analyzed between two groups (saline-treated OIR; metformin-treated OIR). Metformin treatment did not change the extent of avascular areas at P17. However, at P21, when OIR pathology was markedly improved in the saline-treated group, OIR pathology still remained in the metformin-treated OIR group. VEGF expression levels did not differ between metformin- and saline-treated OIR groups at P17 and P21, but Flk1 levels were significantly reduced in the metformin group compared with saline-treated OIR group. Moreover, metformin inhibited VEGF-induced cell proliferation and decreased levels of Flk1 and pFlk1, consistent with the interpretation that metformin inhibits vascular growth by reducing Flk1 levels.

**Conclusion:**

Metformin exerts anti-angiogenesis effects and delays the normal vessel formation in the recovery phase of OIR in mice, likely by suppressing the levels of Flk1.

## Introduction

Blood vessels are formed in two different ways: vasculogenesis, which represents vessel formation from progenitor cells (*de novo* pathway), and angiogenesis, which denotes new vessel formation from pre-existing vessels [1]. Neovascularization is a key pathological contributor not only to tumor growth but also in many retinal diseases. Ischemia may be the central underlying mechanism in retinal diseases involving neovascularization, such as diabetic retinopathy (DR), retinopathy of prematurity (ROP), retinal vein occlusion, retinal arterial occlusion, and ocular ischemic syndrome [2]. DR is a leading cause of blindness and is the most frequently occurring microvascular complication in diabetes [3]. The main causes of vision loss in patients with DR are diabetic macular edema (DME) and diabetic vitreous hemorrhage [3,4]. Vitreous hemorrhage originates from new vessels adjacent to ischemic areas [3,4]. The traditional treatment for DR is laser photocoagulation. Recently, however, vascular endothelial growth factor (VEGF) has been identified as a critical factor for DR and diabetic macular edema. Accordingly, anti-VEGF treatment has shown considerable promise in promoting the regression of new retinal vessels and preventing DR progression [3–5].

ROP is another ischemia-related retinopathy. Normal full-term babies have fully grown retinal vessels at birth. In contrast, premature babies are born with incompletely grown retinal vessels. Higher oxygen saturation can cause retinal vessel growth to shut down, resulting in an avascular ischemic retina. Subsequently, ischemia-triggered neovascularization occurs, followed by vitreous hemorrhage, fibrovascular proliferation, and retinal detachment. Recent studies have demonstrated favorable effects of anti-angiogenic treatment on carefully selected ROP cases [6,7]. A murine model of ROP is oxygen-induced retinopathy (OIR) [8] in which mouse pups are exposed to a high oxygen environment from postnatal day 12 (P12) to P17 and then subsequently switched to a normal oxygen environment. This maneuver causes retinopathy with hypoperfusion and neovascularization that peaks around P17 and disappears by P25 [9].

Metformin, an AMP-activated protein kinase (AMPK) activator, is a widely prescribed anti-hyperglycemic drug for type 2 diabetes [10]. Among anti-hyperglycemic agents, metformin may be especially beneficial in cases of DR [11]. Although the precise mechanisms of metformin action are not yet known, its anti-angiogenic effect [12–16] may contribute to the inhibition of neovascularization in DR and other retinopathies. However, there are conflicting reports in the literature, with some studies describing pro-angiogenic effects of metformin [12,17–19]. In these former cases, a reduction in ischemia rather than a decrease in neovascularization may underlie the beneficial effects of metformin in ischemia-related retinopathy.

Here, we examined the possible beneficial effects of metformin in ischemia-related retinopathy rather than in tumor-related angiogenesis, using OIR. We utilized OIR model to rule out the glucose-lowering effect of metformin on the outcomes of retinopathy. We postulated that if metformin showed anti-angiogenic effect on ischemia-related retinopathy in OIR, we could find possibilities of application of metformin on another type of ischemia-related retinopathy, diabetic retinopathy. To identify possible targets of metformin effects, we followed the time course of changes in OIR.

## Materials and Methods

### Chemicals and reagents

Metformin, cyclohexamide and MG-132 were purchased from Sigma Aldrich (St. Louis, MO, USA). rhVEGF was obtained from R&D Systems (Minneapolis, MN, USA).

### Animals

All mice were treated according to the ARVO statement for the Use of Animals in Ophthalmic and Vision Research, and the study was approved by the Internal Review Board for Animal Experiments of Asan Life Science Institute, University of Ulsan College of Medicine (Seoul, Korea). Pregnant C57BL/6N mice, obtained from Japan SLC, Inc. (Hamamatsu, Japan), were maintained at 24°C ± 0.5°C under a 12-h light/dark cycle. OIR was induced as described previously [8,9]. Newborn mice were reared in a hyperoxic chamber (75% O_2_) from P7 to P12. At P12, they were returned to normoxic room air conditions. Beginning at this time, mice in the metformin group were administered an intraperitoneal injection of metformin (120 mg/kg body weight) until P17 or P21. Mice in the saline-treated group mice were administered an intraperitoneal injection of an equivalent volume of normal saline. To eliminate low growth bias, we excluded any mice weighing less than 6 g at P17 from the study. We classified mice into for groups: Saline-treated control; reared at normal room air from birth to P17 or P21 with intraperitoneal normal saline injection from P12 to P17 or P21, Metformin-treated control; reared at normal room air from birth to P17 or P21 with intraperitoneal metformin injection from P12 to P17 or P21, Saline-treated OIR; OIR induced and saline injection from P12 to P17 or P21, Metformin-treated OIR; OIR induced and metformin injection from P12 to P17 or P21. Mice eyes were harvested after euthanization with carbon dioxide.

### Fluorescein angiography

Fluorescein angiography and fundus images were acquired using a Micron III retinal imaging system (Phoenix Research Laboratories, Inc., Pleasanton, CA, USA). At P17 and P21, mice were anesthetized by isoflurane (1.5%) inhalation, and fluorescein angiography images were obtained after intraperitoneal injection of 0.15 ml of 2% fluorescein sodium (Alcon Laboratories, Inc., Fort Worth, TX, USA).

### Preparation of mouse retina flat mounts

Mouse retina preparation and vessel staining methods were as described by Connor et al. [9], with modifications. Harvested eyeballs were immersed in 4% paraformaldehyde (PFA) at room temperature. After fixing for 1 hour with PFA, sclera, uveal tissue, cornea, and lens were removed from the retina. The isolated retina was washed twice with phosphate buffered saline (PBS) by immersing and gently shaking for 5 minutes. Retinal vessels were stained by storing the retina in Isolectin (B4-594; Molecular Probes, Carlsbad, CA, USA) at 4°C for 2 days. Stained retinal tissues were flat-mounted on glass slides with coverslips.

### OIR grading

Avascular areas were evaluated using Image J (Image processing and analysis tool in JAVA) by two investigators (S. G. Joe and J. A. Choi) blinded to group-identifying information. OIR grade was assessed by two independent, blinded retinal specialists (Y. R. Chung and D. Y. Kim) using the scoring system proposed by Higgins et al. [20] that incorporates assessments of blood vessel growth, blood vessel tufts, extraretinal neovascularization, central vasoconstriction, retinal hemorrhage, and degree of vessel tortuosity.

### Enzyme-linked immunosorbent assay (ELISA) for VEGF

Whole eyes were homogenized in PBS and sonicated. After centrifugation, supernatants containing most soluble proteins were collected. VEGF protein levels in the supernatant were determined using an ELISA kit (Quantikine ELISA mouse VEGF; R&D Systems, Minneapolis, MN, USA). Absorbance values were obtained at 450–570 nm using an Emax spectrophotometer (Molecular Devices, Sunnyvale, CA, USA).

### Cell culture

Human umbilical endothelial cells (HUVECs) and media were purchased from Lonza (Visp, Switzerland). HUVECs were plated on fibronectin-coated plates and cultured in endothelial growth media (EGM) at 37°C in a humidified atmosphere of 5% CO_2_ in air. Cells were used from passages 3 to 9 in the experiments.

### Assessment of cell proliferation

Cells in 6-well plates were placed in a CO_2_ incubator at 37°C for 24 hours in EGM containing 5% fetal bovine serum (FBS) in the absence or presence of 100 ng/mL VEGF (R&D Systems) and VEGF plus 5 mM metformin (Sigma-Aldrich). After 24 hours, cultured cells were detached from the surface of each well by treating with trypsin-EDTA and triturated to yield a single-cell suspension. Cell numbers in each well were quantified using a CyQUANT NF Cell Proliferation Assay kit (Invitrogen, Carlsbad, CA, USA).

### Western blot analysis and immunoprecipitation

Cells were lysed in RIPA buffer (20 mM Tris-Cl pH 7.4, 150 mM NaCl, 1 mM EDTA, 1 mM EGTA, 1% Triton X-100, 2.5 mM sodium pyrophosphate, 1 μM Na_3_VO_4_, 1 μg/ml leupeptin, and 1 mM phenylmethylsulfonyl fluoride). The lysates were centrifuged, and supernatants containing most soluble proteins were collected. Samples containing equal amounts of protein were separated by sodium dodecyl sulfate-polyacrylamide gel electrophoresis (SDS-PAGE) and electroblotted onto polyvinylidene difluoride (PVDF) membranes (Millipore, Bedford, MA, USA) according to standard protocols. Membranes were blocked with 1% bovine serum albumin (BSA) for 30 minutes and then incubated overnight at 4°C with primary antibodies against Flk1 (VEGF-R2, 1:500; Santa Cruz Biotechnology, Santa Cruz, CA, USA), phosphorylated Flk1 (pFlk1; 1:500; Santa Cruz Biotechnology), and α-tubulin (1:2000; Cell Signaling, Beverly, MA, USA). Blots were then incubated for 1 hour at room temperature with horseradish peroxidase-conjugated secondary antibodies, and immunoreactive proteins were detected using enhanced chemiluminescence reagents (Millipore, Billerica, MA, USA). Proteins were quantified by densitometric analysis of band intensities using Image J software.

For immunoprecipitation, cells were lysed in RIPA buffer. Cell lysate was incubated overnight at 4°C with gentle rotation with Flk1 antibody and then precipitated on Protein A and G–agarose beads (Santa Cruz Biotechnology) at 4°C for 2 h. The beads were washed three times with RIPA buffer, boiled in a SDS-PAGE sample buffer for 10 minutes, and centrifuged. Supernatants were subjected to Western blot analysis.

### Flk1 mRNA level of OIR mice and metformin-treated HUVEC

Total RNA was extracted from mouse eye and HUVECs with TRIZOL Reagent (Invitrogen, Carlsbad, CA, USA). cDNA was synthesized from total RNA using a QuantiTect Reverse Transcription Kit (Qiagen, Hilden, Germany). Quantitative polymerase chain reaction (PCR) was performed on a 7300 Real-Time PCR System (Applied Biosystems, Carlsbad, CA, USA) using SYBR Premix Ex Taq Master Mix (Takara Bio, Japan).

### Immunohistochemistry

Eye sections (10 μm thick) prepared using a cryostat was mounted onto glass slides coated with poly-L-lysine. After fixation with 4% PFA, eye sections were permeabilized by incubating in 0.2% Triton X-100 in PBS containing 1% BSA. Fixed, permeabilized eye sections were first incubated with the primary antibody (anti-Flk1, 1:200; Santa Cruz Biotechnology) at 4°C overnight and then with Alexa Fluor-conjugated secondary antibodies (1:500; Invitrogen).

### Statistical analysis

All results are presented as means ± SEM. All statistical tests were performed using SPSS version 15.0 for Windows (SPSS Inc., Chicago, IL). Comparisons of variables between groups were performed employing student’s t-tests or Mann-Whitney U test. Values of P < 0.05 were considered significant. Sigma plot software (version 10.0) was used for all graphical presentations.

## Results

### Effects of metformin on body weight and blood glucose levels of newborn mice

Since metformin is an anti-hyperglycemic drug, it could affect the metabolism of newborn mice. To test this possibility, we measured body weight and blood glucose levels of newborn mice that had undergone the OIR procedure. There was no significant difference in body weight and blood glucose levels between control and metformin-treated groups (not shown), indicating that metformin did not significantly affect body growth and glucose metabolism in newborn OIR mice.

### Metformin inhibited normalization of oxygen-induced retinopathy

Having confirmed that metformin has little metabolic effect on newborn mice, we next assessed the effect of metformin on the development of OIR in these mice. The severity of OIR was quantified by measuring the width of the central avascular zone using Image J and was graded using the OIR grading system.

**Fig 1:**
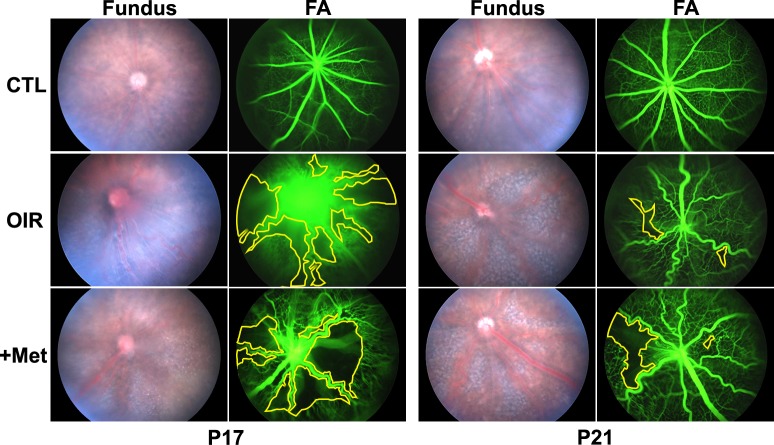
Fundus and fluorescein angiographic images of OIR mice. Control (CTL) mice housed under normal room air oxygen saturation showed fully grown retinal vessels with no avascular areas, tortuous vessels, vessel tufts, or fluorescein leakage at both P17 and P21. Retinas of mice in both saline-treated OIR (OIR) and metformin-treated OIR (+Met) groups exhibited central avascular areas, tortuous vessels, vessel tufts, and fluorescein leakage. At P17, the retinopathy features were similar between the two OIR groups, but at P21, metformin-treated OIR showed poorer vessel growth and diminished retinopathy regression.

**Fig 2:**
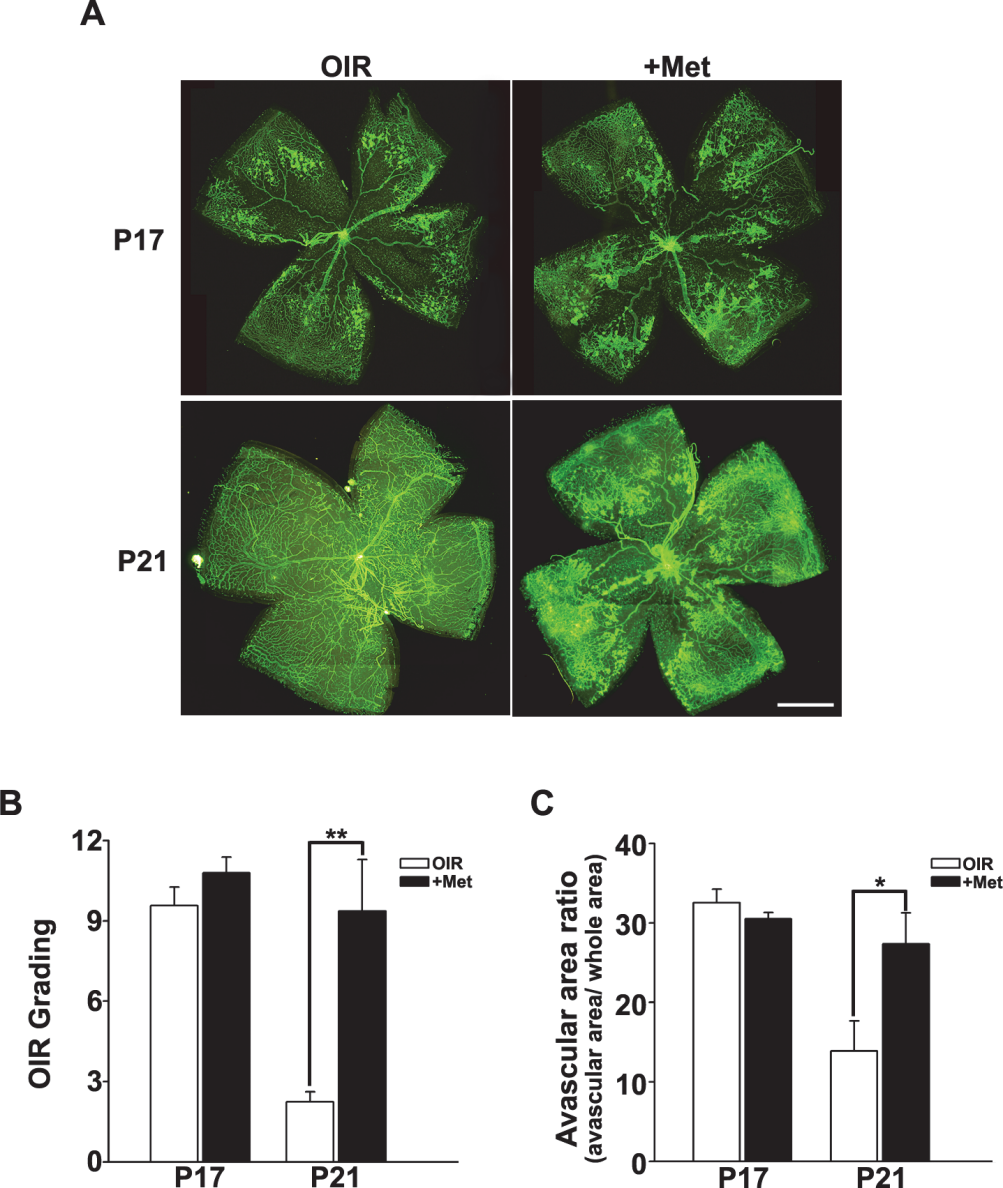
OIR scores and quantification of avascular areas. (A) Flat-mount images showed the same features as were observed in fundus and fluorescein angiographic images (Fig. 1). Both saline-treated OIR (OIR) and metformin-treated OIR (+Met) mice showed central avascular areas, tortuous vessels, and vessel tufts. At P17, retinopathy features were similar between the two OIR groups, but at P21, metformin-treated OIR mice showed poorer vessel growth and diminished retinopathy regression. (B) Retinopathy scores were similar at P17, but metformin-treated OIR mice showed higher scores than OIR mice at P21. (C) Evaluations of avascular areas in the retina revealed no differences between the two OIR groups at P17, but metformin-treated OIR mice exhibited wider avascular areas than saline-treated OIR mice. Original magnification, ×40; scale bar, 100 μm. *P < 0.05, **P < 0.01 (Mann-Whitney U test); P17: n = 7/group, P21: n = 8/group.

At P17, retinas of control mice reared in normal room air (CTL) developed fully grown vessels with no retinal hemorrhaging, vascular tufts, or fluorescein leakage. In contrast, retinas of mice that had undergone the OIR procedure had central avascular zones around the optic disc and exhibited fluorescein leakage from tortuous vessels at P17. At this stage, metformin treatment had no apparent effect on the development of OIR. Avascular areas and OIR scores were not different between saline-treated OIR (OIR) and metformin-treated OIR (+Met) groups (Figs. 1 and 2).

OIR mice substantially revealed regressed pathologic findings at P21, attaining the normal pattern of vessel formation. In contrast, retinas of metformin-treated OIR mice remained abnormal; new vessel formation was substantially reduced and avascular areas were greater than those of saline-treated OIR mice. These findings were confirmed by fluorescein angiography and postmortem flat-mount images, which showed wider avascular zones, more prominent retinal tufts, and tortuous vessels in the metformin-treated group (Figs. 1 and 2). These results suggest that metformin inhibits “catch-up” vessel growth during the regression period in OIR mice.

### Metformin had no effect on VEGF expression

**Fig 3:**
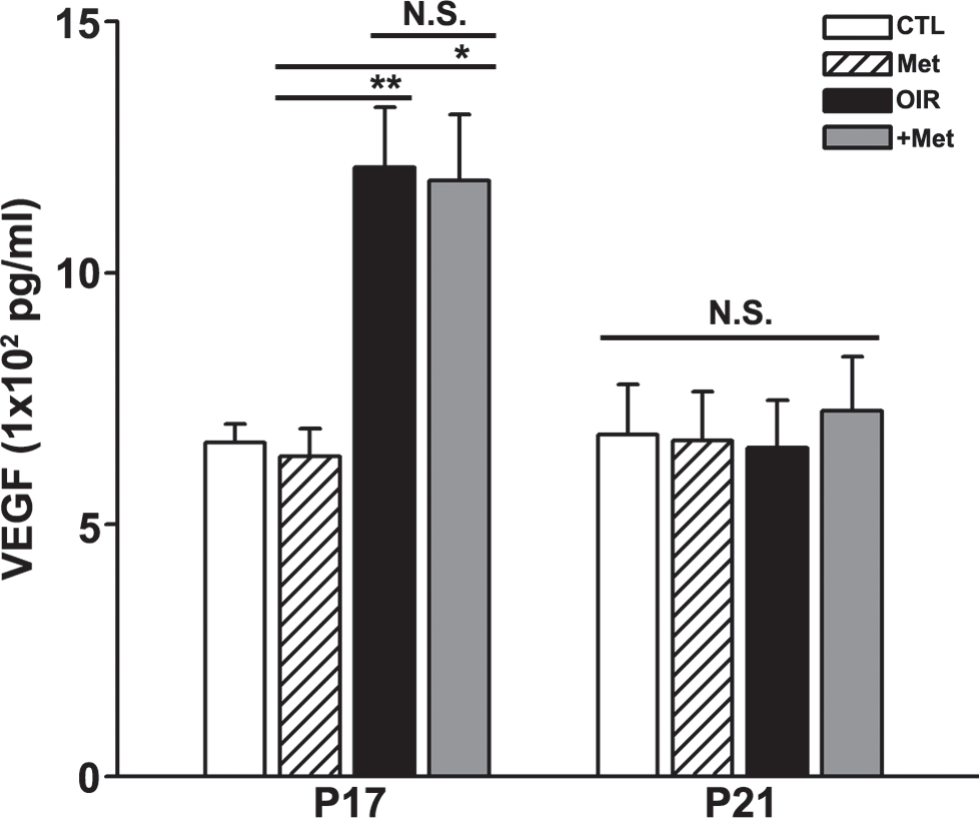
Analysis of VEGF expression level in OIR mouse eyes. ELISAs revealed higher levels of VEGF expression in the eyes of mice in the saline-treated OIR group (OIR; n = 9) and metformin-treated OIR mice (+Met; n = 10) than in those of the control, reared in normoxic condition, (CTL; n = 5) and metformin-treated control (Met; n = 4) group at post natal day 17 (P17). Elevated VEGF levels in both OIR groups decreased at post natal day 21 (P21). But metformin treatment did not affect VEGF level. VEGF levels in metformin-treated OIR mice (+Met; n = 12) were not different from those in saline-treated mice (OIR; n = 12) at P17 or P21. *P < 0.05 and **P < 0.01 compared to metformin-treated CTL (Met; n = 4). (Mann-Whitney U test).

VEGF plays a pivotal role in new vessel formation under both pathological and physiological conditions. In the early stages of OIR, VEGF may contribute to abnormal new vessel formation. However, at least in mice, it may also play a role in the restoration of perfusion during the period of OIR regression. To examine the possibility that the VEGF cascade might be involved in the apparent anti-angiogenic effect of metformin during the period of OIR regression, we measured VEGF levels in whole eyes of OIR and metformin-treated OIR mice by ELISA. Compared to controls mice, reared in normoxic condition from birth to sacrifice (CTL), both saline-treated OIR (OIR) and metformin-treated OIR (+Met) mice eyes showed increased levels of VEGF in the eye at P17 (Fig. 3). At P21, VEGF levels were decreased in both saline-treated OIR and metformin-treated OIR mice eyes (Fig. 3). These results suggest that VEGF production was markedly increased at P17, corresponding to the peak in OIR when retinas may experience a relatively ischemic state through sudden transition from hyperoxia to normoxia. Subsequently, during the restorative period, from P17 through P21, increased levels of VEGF may act to stimulate vessel formation, resulting in a gradual reduction in avascular area, as shown in Figs. 1 and 2. Notably, however, there was no difference in VEGF levels between saline-treated OIR and metformin-treated OIR groups at both P17 and P21. This negative result indicates that the anti-angiogenic effect of metformin was unlikely due to the reduction in VEGF levels. Instead, metformin may affect VEGF receptors or their downstream signaling.

### HUVECs respond to VEGF and metformin

**Fig 4:**
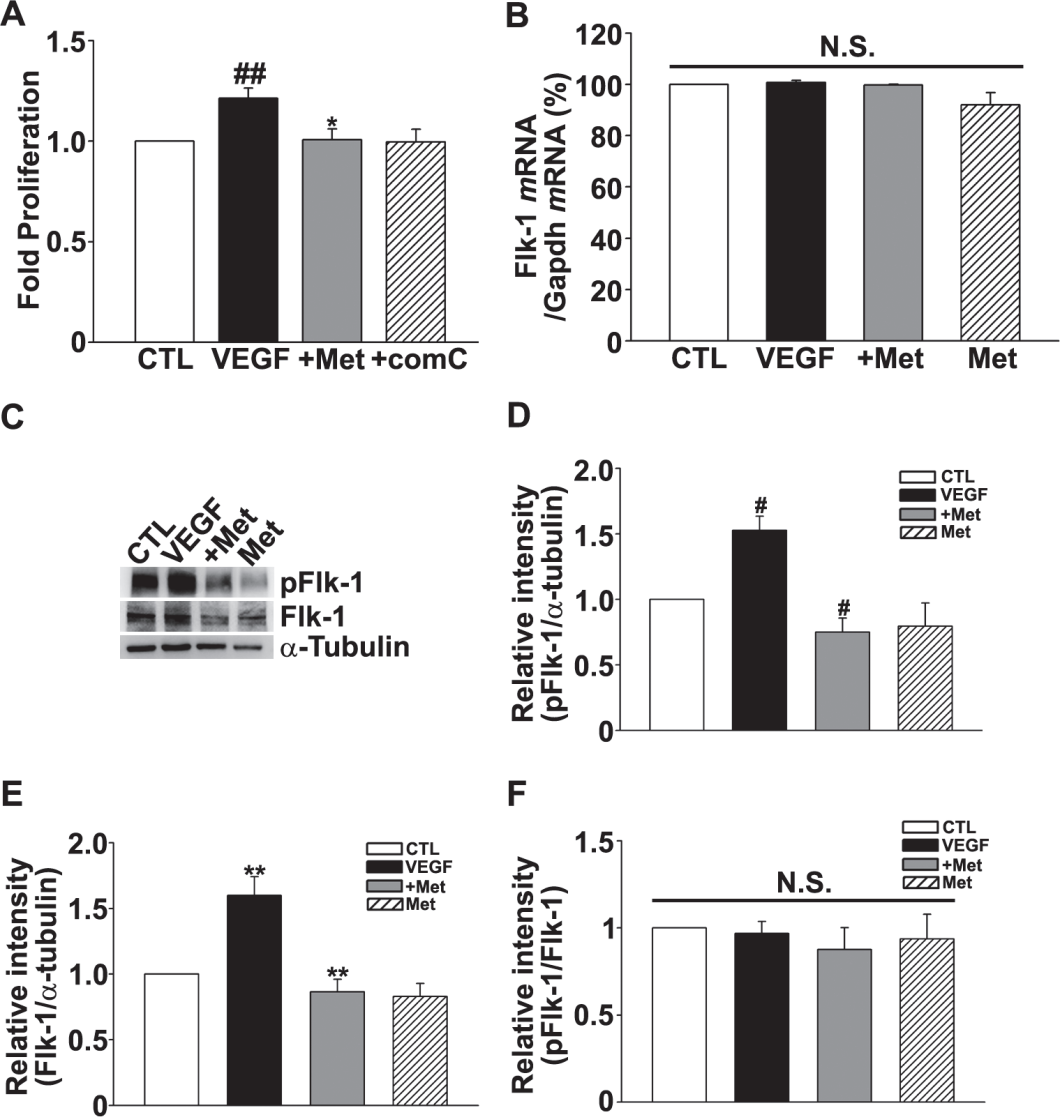
Metformin inhibited VEGF-induced proliferation of HUVECs and reduced Flk1 expression and pFlk1 levels. (A) VEGF-treatment increased the proliferation of HUVECs compared to non-treated control cells (CTL), and metformin inhibited VEGF-induced proliferation. (n = 10, CTL; control HUVECs, VEGF; VEGF-treated HUVECs, +Met; VEGF plus metformin treated HUVECs, Met; metformin-treated HUVECs) (B) RT-PCR data showed that Flk1 mRNA level was not significantly changed in HUVECs treated with VEGF or VEGF plus metformin (n = 3). (C) Western blotting for pFlk1 and Flk1 in HUVECs after exposed to VEGF or VEGF plus metformin (n = 4). (D, E and F) Densitometric analysis of Western blots showed that VEGF increased the levels of pFlk1 and Flk1 in HUVECs, and demonstrated that metformin inhibited these increases. *P < 0.05, **P < 0.01, ^#^P < 0.005 and ^##^P < 0.001.

HUVECs express the VEGF receptors, Flk1 (VEGFR2) and Flt1 (VEGFR1), and proliferate in response to VEGF administration. Thus, we used HUVEC proliferation as a read-out to examine metformin effects on VEGFR-related processes. As expected, a 24-hour treatment with VEGF (100 ng/ml) increased the number of HUVECs compared with unstimulated controls. Addition of metformin attenuated the VEGF-induced proliferation of HUVECs, suggesting that metformin reduced the sensitivity of HUVECs to a given amount of VEGF (Fig. 4A). This result is consistent with the above hypothesis that either VEGFRs or downstream signaling is affected by metformin.

### Reduction in Flk1 by metformin

Of the two types of VEGF receptors, Flk1 is considered the main mediator of the angiogenic effects of VEGF [21]. Therefore, we first investigated whether treatment with metformin altered the expression of Flk1 by evaluating Flk1 mRNA and protein levels in control and metformin-treated HUVECs. Notably, VEGF treatment increased the protein levels of both Flk1 and its activated form pFlk1 (Fig. 4C), but did not change Flk1 mRNA levels (Fig. 4B). Metformin also substantially attenuated VEGF-induced increases in Flk1 and pFlk1 protein levels (Fig. 4C-E).

**Fig 5:**
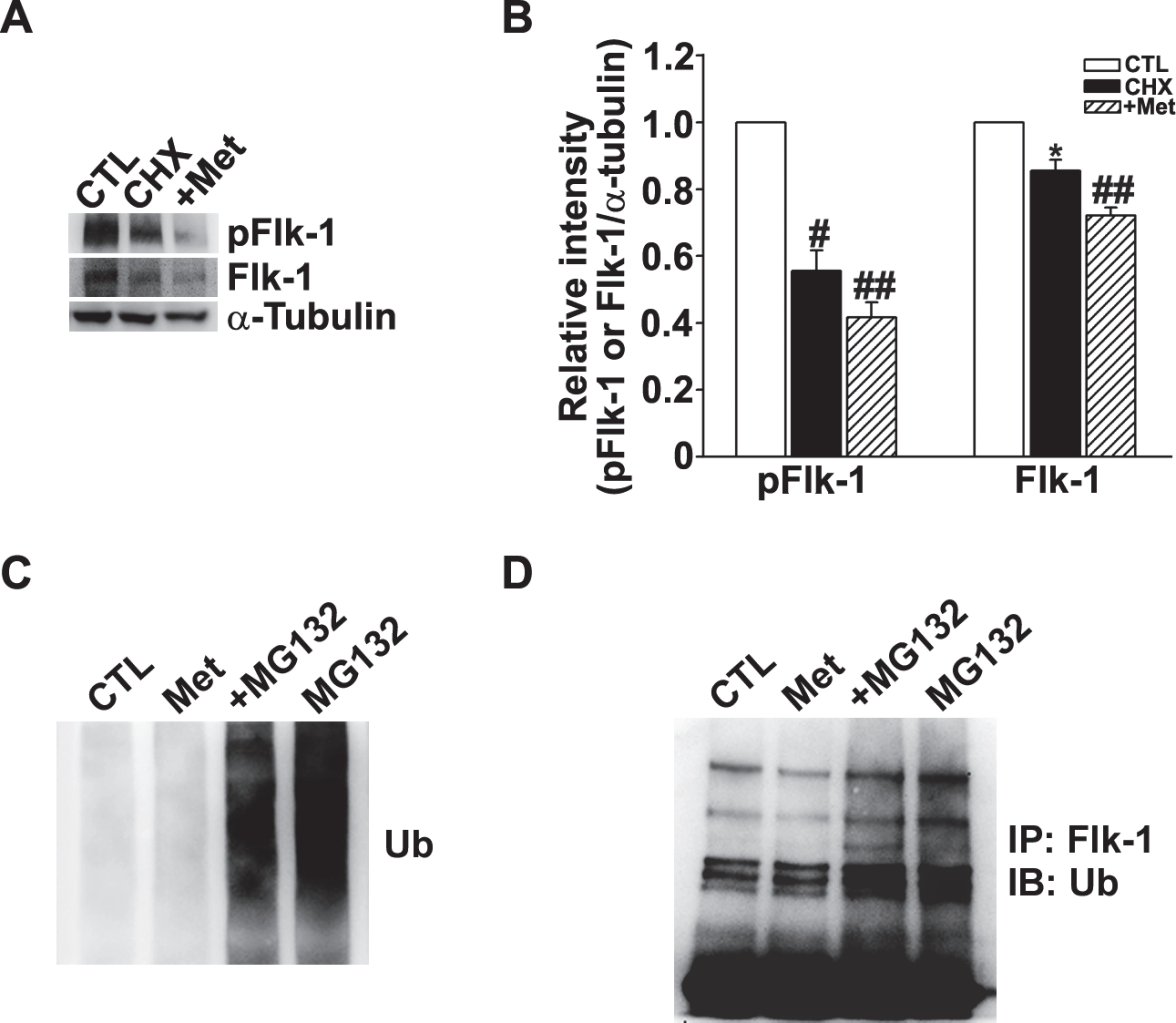
Metformin reduced the level of Flk1 through inhibition of its degradation by ubiquitin proteasome system (UPS). (A) Western blotting for pFlk1 and Flk1 in HUVECs after exposed to cycloheximide (CHX) or CHX plus metformin (n = 3). Protein synthesis was blocked by CHX treatment, and the addition of metformin further reduced both pFlk1 and total Flk1 protein levels. (B) Densitometric analysis. (C) Western blotting for anti-ubiquitin (Ub) in HUVECs after treatment with metformin or metformin plus MG-132, a potent inhibitor of the UPS (n = 4). (D) Immunoprecipitation (IP) with anti-Flk1 antibody, followed by western blotting (IB) with anti-Ub (n = 3). Metformin reduced the ubiquitination of Flk1, which was reversed by MG-132. *P < 0.05, ^#^P < 0.005 and ^##^P < 0.001.

Since the pFlk/Flk ratio did not change, the above findings indicated that metformin did not reduce Flk1 activation per se but rather decreased the total level of Flk1 (Fig. 4F). To further investigate whether metformin reduces the level of Flk1 by inhibiting its protein synthesis, we treated HUVECs with the protein synthesis inhibitor cycloheximide (CHX), with or without the addition of metformin. Even when overall protein synthesis was almost completely blocked by CHX treatment, the addition of metformin further reduced both pFlk1 and total Flk1 protein levels (Fig. 5A and B), indicating that metformin down-regulates Flk1 by a mechanism other than inhibition of protein synthesis. One possible mechanism of protein synthesis inhibition might be through protein-degrading ubiquitin-proteasome system (UPS). We tested this hypothesis by treating HUVECs with metformin and MG-132, a potent inhibitor of the UPS; we found that metformin-induced downregulation of Flk1 and its ubiquitination, was reversed by co-treatment with MG-132, indicating that the effect of metformin on Flk1 was partially dependent on the activation of the UPS (Fig. 5C and D). In contrast, metformin did not seem to significantly activate autophagy, another protein degradation pathway, since it failed to increase the level of LC3-II (not shown).

**Fig 6:**
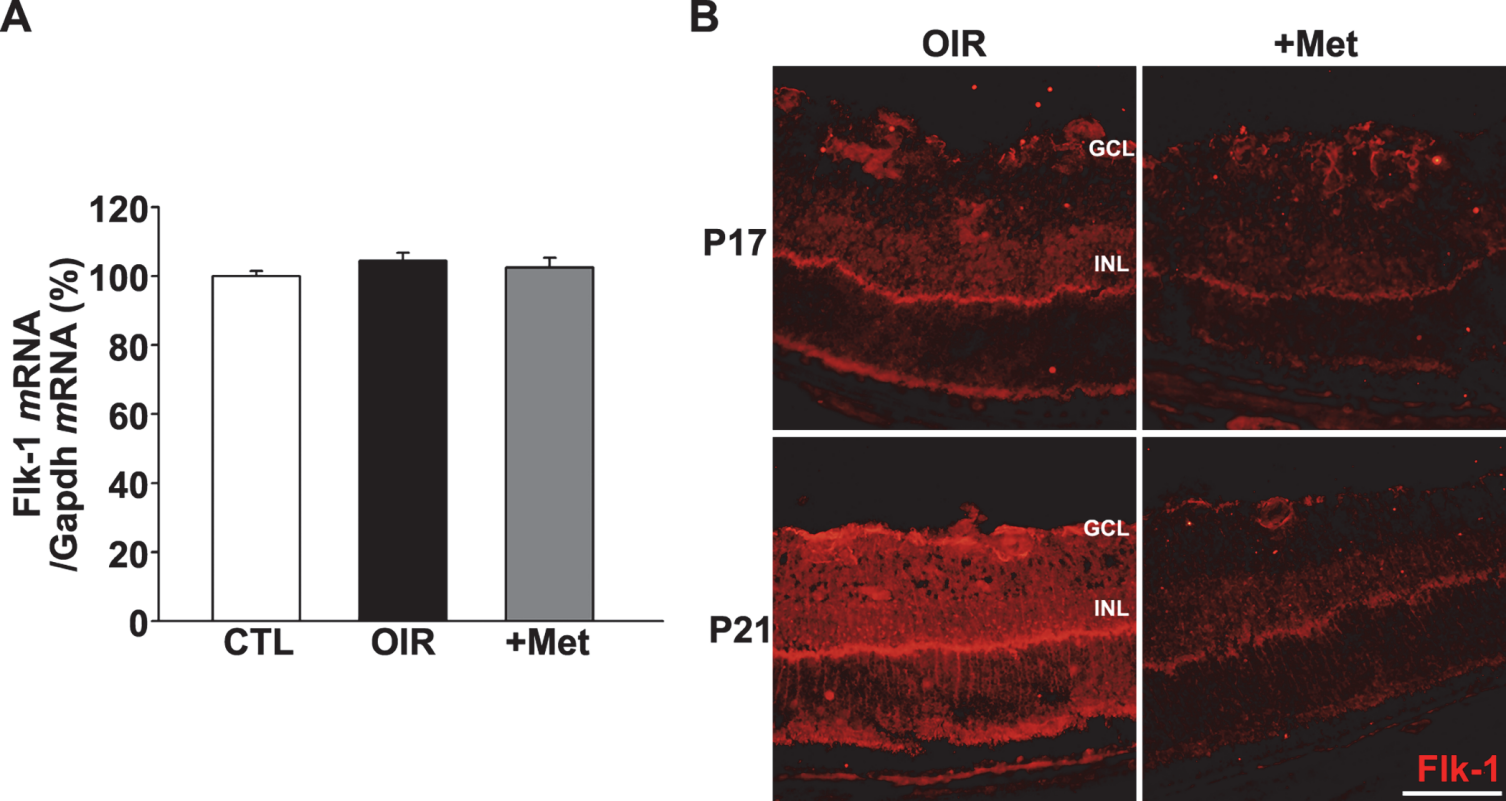
Metformin reduced the level of Flk1 in OIR mouse retinas. Flk1 expression in mouse eyes was quantified using RT-PCR and in retinal section with immunostaining. (A) RT-PCR data showed that Flk1 mRNA levels were not significantly changed in retinas of saline-treated OIR (OIR) or metformin-treated OIR (+Met) mice (n = 6). (B) Immunostaining showed a decrease in Flk1 expression in metformin-treated OIR mice (+Met) compared to saline-treated OIR (OIR) mice at P17 and P21. GCL; ganglion cell layer, INL; inner nuclear layer. Original magnification, ×400; scale bar, 100 μm.

We observed similar changes in the retina of OIR mice as well. Whereas metformin treatment did not change Flk1 mRNA levels in the retina of OIR mice (Fig. 6A), immunostaining revealed that Flk1 protein levels were decreased in the retina of metformin-treated OIR mice at P17 and P21 (Fig. 6B).

## Discussion

A growing body of evidence indicates that the anti-hyperglycemic drug metformin, an AMPK activator, may have diverse beneficial effects beyond its glucose-lowering activity, not only in diabetes but also in other diseases involving new vessel formation. For instance, metformin may reduce complications associated with diabetic retinopathy, such as vitreous hemorrhage [22]. In addition, it may decrease neovascularization in the context of cancer metastasis and growth [23,24].

Diabetic retinopathy is a leading cause of blindness in the adult population. Investigations into the mechanism underlying diabetic retinopathy and studies designed to assess the potential beneficial effects of possible treatments may require animal models such as the murine streptozotocin model. However, because metformin likely affects blood glucose levels per se, it may be difficult to dissect out metformin actions unrelated to its effects on glucose levels using this model. Hence, in the present study, we turned to OIR, another model of ischemic retinopathy. The central finding of the present study is that metformin significantly inhibited new vessel formation during the restorative period following OIR formation. This effect is unlikely due to metabolic actions, since metformin treatment did not alter blood glucose levels or body weights of newborn mice without diabetes. In addition, results obtained from cultured HUVECs as well as OIR mice showed that the underlying mechanism of the anti-angiogenesis effect of metformin likely involves decreasing the level of Flk1, the major receptor for VEGF.

OIR is an animal model for human ROP, a condition involving abnormal neovascularization and hemorrhage. Mouse OIR progresses through several phases: 1) vasoconstriction during exposure to a hyperoxic environment (P7-P12); 2) fulminant OIR after transition to a normoxic environment, presumably caused by relative hypoxia and increased VEGF expression (P12-P17); and 3) a restorative phase, involving development of normal vessels and a reduction in avascular areas (P17 onwards). In the present study, metformin was given at P12 (the beginning of phase 2) through P17 or P21. In this experimental paradigm, metformin did not reduce OIR severity assessed at P17; the size of avascular area, fluorescein leakage, and vessel tortuosity were not different between saline-treated OIR and metformin-treated OIR groups. In contrast, during the restorative phase, metformin treatment markedly retarded the normalization of OIR. Since the restoration of OIR also involves new vessel formation into the avascular area, it is likely that metformin acts to inhibit angiogenesis at this phase.

The current consensus is that VEGF plays a key role in the progression of ischemia-related retinopathy. The progression of hypoperfused and avascular retinal areas in both diabetic and ROP eyes is correlated with an increase in VEGF levels [2,6,25,26]. Increased VEGF expression leads to new immature vessel formation, which could lead to leakage and fibrovascular proliferation. Consequently, anti-VEGF treatments such as bevacizumab decrease ROP by inhibiting new vessel formation and fibrovascular proliferation [6]. Since decreased formation of vessels under hyperoxia and consequent hypoperfusion under normoxia may be the mechanism of ROP, early stimulation of angiogenesis with intravitreal injection of angiopoietin-1 during the early ROP period could inhibit ROP progression and induce normal vessel growth [27]. In contrast, anti-VEGF treatment at the time of peak ROP was reported to inhibit abnormal new vessel growth in ROP [6], and was thus beneficial. Hence, depending on the phase of ROP and the particular angiogenic stimulus, both angiogenic and anti-angiogenic drugs may be therapeutic in ROP and possibly in other retinopathies.

Considering the prominent role of VEGF in angiogenesis, the apparent anti-angiogenic effect of metformin during the restorative phase of OIR may also involve changes in certain steps of VEGF signaling. VEGF exerts its angiogenic effects by binding to VEGF receptors (VEGFRs) [2,28,29]. There are three VEGFRs: VEGFR-1 (Flt1), VEGFR-2 (Flk1/KDR), and VEGFR-3 (Flt4). VEGFR-1 and VEGFR-2 are associated with angiogenesis and VEGFR-3 is associated with lymphangiogenesis [29]. Among VEGF receptors, Flk1 is known to be the main angiogenic receptor [28]. In this study, we evaluated the level of Flk1 and pFlk1 in HUVECs using Western blotting. Treatment of HUVECs with VEGF increased the expression of Flk1 and pFlk1 compared to unstimulated control cells. However, VEGF plus metformin or metformin alone decreased expression of Flk1 and pFlk1 compared to HUVECs treated with VEGF or even to control HUVECs. These results lead to the conclusion that metformin inhibits the induction of Flk1 by VEGF in HUVECs. Since metformin did not alter the pFlk1/Flk1 ratio, it may not affect the VEGF-Flk1 interaction per se. Metformin seems to down-regulate Flk1 and pFlk1 mainly through the increased degradation via UPS. Consistent with these *in vitro* results, immunostaining revealed that metformin markedly reduced Flk1 levels in the retinas of OIR mice.

Metformin is a biguanide AMPK activator. It is widely used as an anti-diabetic drug because it controls blood glucose levels by increasing insulin sensitivity and enhancing glucose uptake in the liver [30]. Metformin has recently been reported to have other actions, such as anti-cancer effects and regulation of the unfolded protein response [31–34]. Anti-cancer effects were originally revealed by cohort studies, which showed that the incidence of cancer was lower in diabetic patients taking metformin [12,16,35–37]. In some cases, the anti-angiogenic effects of metformin may underlie its anticancer effects [12,14–16]. Esfahanian et al. [16] showed that metformin substantially inhibits proliferation, migration, and MMP-2 and -9 expressions in HUVECs, an effect that is partially dependent on AMPK. Dallaglio et al. [12] found that metformin inhibits VEGF-dependent activation of extracellular signal-regulated kinase 1/2. Metformin also lowers VEGF levels in diabetic patients [38]. However, in the current study, metformin did not affect VEGF expression in OIR mouse eyes. Instead, our results indicate that metformin down-regulates Flk1, the main receptor for VEGF.

It is well known that treatment with anti-VEGF agents such as bevacizumab attenuates ROP and OIR progression, and paradoxically induces normal vascular growth into the avascular retina [6,7]. Increased VEGF in ischemic retinas, such as those in ROP or diabetic retinopathy, can induce neovascularization, which is inhibited by anti-VEGF treatment. In the current study, metformin also showed an anti-angiogenic effect, likely through down-regulation of Flk1 in the retina. Although this effect—the delayed normalization of OIR—is obviously not desirable in this particular model, the anti-VEGF or anti-angiogenic effect of metformin might have beneficial effect on proliferative diabetic retinopathy, in which neovascularization is the core pathology. In this regard, metformin may be the first drug reported to directly down-regulate Flk1. Further studies will be required to elucidate the specific mechanism involved in this effect of metformin.
